# The Relationship Between Assistance Satisfaction and Negative Affect in Long-Term Social Assistance Recipients in China: The Moderating Role of Self-Acceptance

**DOI:** 10.3389/fpsyg.2019.00109

**Published:** 2019-01-31

**Authors:** Xinru Huang, Hong Chen, Shanshan Li

**Affiliations:** School of Management, China University of Mining and Technology, Xuzhou, China

**Keywords:** assistance satisfaction, governing subjects, negative affect, self-acceptance, moderating effect, long-term social assistance recipient

## Abstract

Public welfare in China is less universal, comprehensive, and generous when compared with other welfare regimes, especially for individuals with occupational disease. The assistance satisfaction of Chinese pneumoconiosis patients, a typical group of long-term social assistance recipients, has been linked to diminished health and psychological well-being. Self-acceptance is theorized to promote well-being, which may buffer the negative consequences of negative emotion on assistance satisfaction. This research was conducted based on the survey data of 1,345 patients in typical mining areas of China. In addition to single-factor analysis of variance, descriptive analysis, and correlation analysis, a cluster analysis was performed to explore the profiles of participants' ratings of assistance satisfaction with five governing subjects: government, employing unit, medical institution, welfare organization, and laborer themselves. The results were as follows: (1) Social assistance satisfaction perceived by Chinese long-term social assistance recipients was at an average level, wherein self-assistance satisfaction perception was the lowest among five dimensions. (2) The overall perception of assistance satisfaction of Chinese long-term social assistance recipients was significantly correlated with educational background and household monthly income; specifically, the participants with higher levels of education and lower levels of income were more likely to have higher assistance satisfaction perception. (3) Cross analysis showed that participants with higher assistance satisfaction were mainly from the low-high group, having a lower level of negative affect, and higher level of self-acceptance. (4) The average negative affect level was above the scale midpoint (3.65) and negatively associated with each of the assistance satisfaction ratings. (5) The analysis yielded three distinct profiles—*medium* (19.26%), *high* (40%), and *low* (40.74%)—according to their degree of assistance satisfaction. Relative to the other profiles, low assistance satisfaction participants reported greater levels of negative affect in their daily life. However, self-acceptance moderated these effects, but no moderating effect was detected for individuals reporting greater levels of assistance satisfactory. Purpose is proved empirically a positive asset for promoting psychological adjustment in the period of receiving social assistance for Chinese long-term social assistance recipients.

## Introduction

China' public welfare is often considered less universal, comprehensive and generous when compared with other European countries. This view can often be explained that Chinese residents can hardly enjoy the benefits from the current welfare regime due to weak welfare system, inefficient markets, and unstable politics. As a matter of fact, Chinese government has made great efforts to provide a safety net to prevent people from suffering the effects of poverty and further to strengthen the building of welfare facilities. However, Chinese government has not ever reached original desired governance effect until now, due to its less developed economy and industrialization compared with other developed economies (Flora and Heidenheimer, 1981; McLaughlin, 1993; Holliday, 2000). In China, all citizens are entitled to obtain their primary income from social insurance through a maintenance system for citizens who fulfill specific criteria. One crucial criterion for social insurance benefits is determined by a citizen's work record. Specifically, rights to receive social insurance (e.g., pension insurance, medical insurance, unemployment insurance, and occupational insurance) are obtained by labor market participation. When people have no right to receive social insurance and no ability to support themselves, they are eligible for social assistance (Loyland, 2016). Social assistance refers to the system in which the state and society provide financial assistance and life support to the citizens who are financially trapped for various reasons. Social assistance, unlike social insurance, as an integral part of the social security system, has different social security goals. The goal of social insurance is to prevent labor risk, while the goal of social assistance is to alleviate the difficulty of life. Pneumoconiosis patients are a typical group of long-term social assistance recipients (LTRs), who have failed to obtain social insurance for work-related injury identification due to various reasons and thus depending on social assistance as their sole source of income.

Pneumoconiosis is currently the most serious occupational disease in China, accounting for about 80% of individuals with work-related injury (van Oorschot, 2006). In recent years, pneumoconiosis has shown an increasing trend, growing at a rate of 26,000 cases per year (Crawford and Henry, 2004). Occupational disease is one type of occupational injury. When a worker is diagnosed with occupational disease, he or she shall have an occupational injury identification and appraisal of labor capacity to decide what occupational injury insurance treatment he or she should receive. However, most patients fail to obtain work-related injury certification due to lacking a labor contract and other certifications provided by employers, because these persons are mainly migrant workers working illegally and most employees had left their workplace for many years after being diagnosed with occupational disease and thus failing to contact their employers. Therefore, the majority of pneumoconiosis patients are LTRs, defined as people who have received social assistance as their main source of income for at least 6 of the last 12 months. More importantly, LTRs often struggle with numerous health issues (e.g., poorer functional health, pain, depression, and anxiety) and consequently have low levels of subjective well-being (Taylor and Barusch, 2004; Vozoris and Tarasuk, 2004; Kaplan et al., 2005). Life satisfaction is regarded as an indispensable component of subjective well-being (Diener et al., 2012). Perceived economic status is positively related to life satisfaction (Camfield and Esposito, 2014), and social assistance provides an economic safety net and is a means-tested benefit. Therefore, assistance satisfaction (AS) plays a more significant role among LTRs than other groups. The Law of Occupational Disease Prevention and Treatment of the People's Republic of China issued by the NPC Standing Committee in 2017 provides that “occupational disease prevention work upholds the policy of prevention first, combining prevention and treatment, establishing a mechanism of the employer taking responsibility, administrative agency supervising, industry self-disciplining, employee participating, and society supervising.” From the perspective of the collaborative governance of occupational safety and health, the present research measured assistance satisfaction with five governing subjects, respectively: government (GAS), employing unit (EAS), medical institution (MAS), welfare organization (WAS), and the effects of self-assistance (SAS) (Shi et al., 2015).

Apart from the noted negative experience of LTRs, there exists some empirical evidence proving that people in negative mood tend to report lower ratings of satisfaction than people in positive mood (Abele and Gendolla, 1999; Diener et al., 2012). Thus, identifying which factors could affect individuals' AS ratings may deepen our understanding of satisfaction adjustment. One such factor may be sense of self-acceptance. Acceptance concern has been discussed as a foundation for the satisfaction judgments of disadvantaged/vulnerable groups (Hu et al., 2012) that may help themselves incorporate perceived self-acceptance into a coherent understanding of who they are. To date, however, the extent to which self-acceptance interacts with different AS ratings has not been explored. In the current report, we aim to provide an initial test of this issue by (a) identifying the unique profiles of LTRs in terms of AS ratings and its dimensions (taking individuals with work-related injury as subjects) and investigating their associations with indicators of negative affect (NA), and (b) examining self-acceptance as moderator of the emotion–AS perception relationship.

## Literature Review

### The Nature of Social Assistance and Its Correlation With Negative Affect

A welfare state is defined as a state with the responsibility for securing some basic modicum of welfare for its citizens (Esping-Andersen, 1990). In European welfare states, Europeans share a common and fundamental deservingness culture, which holds that the elderly, sick, and disabled deserve to receive more welfare provisions than unemployed people or immigrants (Crawford and Henry, 2004). However, views on those who deserves or does not deserve the assistance of society may differ among individuals, which may influence the recipients' own emotional experience of living on social assistance (Marttila et al., 2010). In addition to having limited income, the experiences of LTRs living on social assistance may be negatively impacted by other factors, such as loss of autonomy and being dependent on social services, feeling shame, and not being part of society (Starrin and Kalander, 2001; Underlid, 2006; Weinberg, 2016), which makes LTRs more likely to have negative emotions such as psychological and mental distress, anxiety, and depression (Malmberg-Heimonen, 2011; Loyland, 2016). Negative experiences have been demonstrated to have a causal influence on satisfaction judgments (Schwarz and Clore, 1983), including life satisfaction, compassion satisfaction, and job satisfaction (Joshanloo, 2016; Ridgway and Clayton, 2016; Sadri and Ali, 2016).

Still unknown, however, is whether average NA level exerts a relationship with AS perception and its dimensions. More recent empirical evidence on negative consequences has emphasized satisfaction judgements regarding jobs, compassion, social support, and overall life (Krause, 1995; Shaw et al., 1999; Manjrekar and Berenbaum, 2012; Craigie et al., 2016; Joshanloo, 2016) along with present negative outcomes. Satisfaction with social support can be determined by the amount of assistance provided by significant others; this receiving process produces negative interactions (Krause, 1995). Specifically, Razurel and Kaiser (2015) developed a satisfaction scale for social support to investigate the relationship of social support satisfaction with the psychological health of primiparous mothers in terms of depressive symptoms, anxiety, and parental self-efficacy; this satisfaction scale revealed five sources of support: the spouse, the young woman's mother, family, friends, and professionals. Thus, NA may pose significant challenges to psychological health, thus reducing the levels of AS perception.

### Self-Acceptance as a Moderator of the Effects of Social Assistance Satisfaction

There is indication that emotional well-being can explain LTRs' AS perception more objectively, while individual differences may affect the magnitude of this association. A prominent factor that may shape the relationship of AS perception relate to adjustment is self-acceptance. Self-acceptance is theorized to be affirmation or acceptance of oneself in spite of his/her weaknesses or deficiencies (Mearns, 1989), which has routinely been shown to be related to positive outcomes (Chamberlain and Haaga, 2001). On the contrary, the absence of ability to unconditionally accept oneself can lead to various emotional difficulties (e.g., uncontrolled anger and depression) (Carson and Langer, 2006). As the preceding theoretical argument indicates that the high level NA and LTRs' inherent nature may render participants vulnerable to experiencing low levels of psychological well-being and to feeling that they belong to the disadvantaged group in society, in this context, feelings of self-acceptance seems to be more important and they are eager to receive approval, respect, or love from other people (Lundh, 2004). Individuals may present different profiles of AS ratings regardless of their level of self-acceptance, but those with a greater sense of self-acceptance may interpret such ratings within a broader and perhaps more personally meaningful context. Thus, self-acceptance may be a third-order source of resilience to the negative experience of being an LTRs.

As a framework for understanding the experience of LTRs, self-acceptance may also inherently decouple the link between negative mood and AS perception in other words. Like the gear lubricant that can make machines run more smoothly, self-acceptance may dampen the effects of negative emotional experiences, thus improving the AS perception caused be psychological distress in everyday life. Indeed, there is some empirical support for the moderating role of self-acceptance in the relationship between NA and AS. Among university students, studies showed that the positive link between mindfulness and subjective well-being was significantly mediated by self-acceptance (Xu et al., 2016). Therefore, whether self-acceptance interacts with NA to influence the experience of LTRs needs more empirical research study.

## Methods

### Participants and Procedure

Pre-investigation was conducted in April 2017. Pneumoconiosis sufferers at Xuzhou Mining Group General Hospital were selected as interviewees for pre-investigation. We distributed 350 questionnaires on site, recovering 322 questionnaires. After eliminating invalid questionnaires, 290 valid questionnaires were obtained. [The research of the pneumoconiosis patients was approved by the Ethics Committee of General Hospital of Xuzhou Mining Group on the March 18th, 2017 (Project Identification Code: 2017031802)]. Data were analyzed using the leakage value test, descriptive statistics test, extreme group comparison, and homogeneity test. After the deletion of items with load value < 0.5 and revision of the remaining items, the official questionnaire was formed.

Formal investigation was conducted from May to July 2017. In addition to the Xuzhou mining area selected during preliminary investigation, we also selected large-scale coal bases in the Sanjin (Shanxi), Lianghuai (Anhui), and central plain (Henan, Hebei) regions as research areas based on their reserves, types, quality of coal, exploiting conditions, types of damage, and location characteristics. Investigation was mainly carried out at locations that had many workers with work-related injury, such as occupational disease prevention institutes and miner general hospitals of the related mining area. This descriptive, cross-sectional study surveyed people receiving long-term social assistance in China about their health and social functioning. LTRs were included in this study if they had received social assistance as their main source of income for at least 6 of the last 12 months, and were able to complete the study questionnaire. Similar to the preliminary investigation, before distributing the questionnaire, subjects were selected through stratified sampling to cover patients of different regions, genders, ages, educational backgrounds, household income, number of children, working years, length of stay, and pneumoconiosis phases to ensure a scientific, diverse, and representative sample (Table 1). Researchers distributed 2,000 questionnaires mainly via site investigation during the scheduled period of 3 months, recovering 1,673 questionnaires (recovery rate of 83.65%). After questionnaires were recovered, low-quality questionnaires were reviewed and eliminated by special personnel. A total of 1,345 effective questionnaires were obtained, an effective rate of 80.39%. [All the investigating procedures in this research were performed in accordance with the relevant guidelines and regulations of the Ethics Committee of all the related institutions].

**Table 1:** Sample structure of the questionnaire (*N* = 1345).

**Variables**		**Number of participants (%)**	**Variables**		**Frequency (%)**
Gender	Male	1340 (99.63%)	Accumulated dust exposure age (year)	< 10	409 (30.41%) 317 (23.57%)
	Female	5 (0.37%)		10–15	
Age (years)	< 40	281 (20.89%)		16–20	319 (23.72%)
	41–50	431 (30.04%)		21–25	215 (15.99%)
	51–60	449 (33.38%)		>25	85 (6.32%)
	61–70	180 (13.38%)		>20	15 (1.12%)
	>70	4 (0.29%)		< 2000	84 (6.25%)
Educational level	illiteracy	38 (2.83%)	Household monthly income (yuan)	2000–4000	252 (18.74%)
	Primary school	178 (13.23%)		4001–6000	127 (9.44%)
	Junior middle school	602 (44.76%)		6001–8000	781 (58.07%)
	Senior high school/vocational school	527 (39.18%)		8001–10000	101 (7.51%)
Length of stay	< 5	56 (0.16%)	Number of children	0	151 (11.27%)
	6–10	197 (14.65%)		1	640 (47.58%)
	11–15	618 (45.95%)		2	200 (14.87%)
	>15	474 (35.24%)		3	339 (25.20%)
Pneumoconiosis phase	pneumoconiosis I	782 (58.14%)		4	15 (1.12%)
	pneumoconiosis II	415 (30.86%			
	pneumoconiosis III	148 (11.00%)			

### Measures

#### Social Assistance Satisfaction

Participants were asked to rate their social assistance satisfaction using five items, each focused on a different form of social assistance: (a) “After suffering disease, I am very satisfied with the assistance from the government.”; (b) “After suffering disease, I am very satisfied with the assistance from the employing unit”; (c) “After suffering disease, I am very satisfied with the assistance from the medical institution”; (d) “After suffering disease, I am very satisfied with the assistance from the welfare organization.” Additionally, participants were asked to rate the statement (e) “After suffering disease, I am very satisfied with the results from assisting myself through self-safeguarding my own deserved legal rights.” Responses ranged from 1 (strongly disagree) to 5 (strongly agree).

#### Negative Affect

Negative affect (NA) was measured using 10 negative items from the Positive and Negative Affect Schedule (PANAS) (Watson et al., 1988). As pointed out by Crawford and Henry (2004), the originally designed NA items from the PANAS measure only measures negative emotions with higher-level subjective distress and unpleasurable engagement, in the absence of lower-level negative emotions. Therefore, the original 10 items were supplemented with five lower-arousal negative emotions, including frustrated, down, sad, grouchy, and anxious. Participants indicated their experience of NA over the past week on a scale ranging from 1 (not at all) to 5 (extremely). The PANAS has good reliability and validity in the field of affective well-being (Watson et al., 1988). Additionally, previous research demonstrated good reliability and internal consistency of the 10 NA items among UK residents (Crawford and Henry, 2004).

#### Self-Acceptance

Self-acceptance was assessed using the selected subscale from the Ryff Scales of Psychological Well-Being (Ryff, 1989), which is designed to assess the positive attitude toward oneself and one's past life (Springer and Hauser, 2006). The self-acceptance scale was found to have factorial and content validity among psychology student, professional, and household samples (Kafka and Kozma, 2002; van Dierendonck, 2005; Springer and Hauser, 2006). Sample items include “In general, I feel confident and positive about myself,” “When I compare myself to friends and acquaintances, it makes me feel good about who I am,” and “In many ways, I feel disappointed about my achievements in life” (reverse scored). Response choices ranged from 1 (strongly disagree) to 5 (strongly agree).

## Results

### Difference Analysis

We analyzed the differences in AS perception and its dimensions as well as the differences in NA and self-acceptance across demographic variables, household variables, and length of stay using the independent samples *t*-test and one-way ANOVA (Table 2).

**Table 2:** One-way ANOVA analysis.

		**AS**	**GAS**	**EAS**	**MAS**	**WAS**	**SAS**	**NA**	**Self-Acceptance**
Age	F	1.516	1.601	1.752	3.350^**^	0.095	0.878	1.033	1.458
Educational level	F	8.429^***^	3.303^**^	5.145^***^	12.476^***^	2.195^**^	2.443^**^	6.688^***^	1.091
Household monthly income	F	4.860^***^	2.255^**^	6.986^***^	4.084^**^	1.667	3.363^**^	5.149^***^	1.472
Number of children	F	1.883	0.469	4.809^***^	5.606^***^	0.865	1.296	1.310	0.089
Length of stay	F	1.581	2.018	2.805^**^	3.894^**^	0.900	3.047^**^	3.520^**^	0.259

*** p < 0.01;

** *p < 0.05*.

As seen in Table 2, MAS varied significantly with age: AS and its dimensions varied significantly with educational background; AS, GAS, EAS, MAS, SAS, and NA varied significantly with household monthly income; EAS and MAS varied significantly with number of children and length of stay; SAS and NA varied significantly with length of stay. Self-acceptance did not vary significantly with age, educational background, household monthly income, number of children, or length of stay. Thus, AS perception only varied significantly with educational background and household monthly income.

### Descriptive Statistical Analysis

Table 3 shows that the mean value of LTRs' AS was at an average level, and nearly half of participants expressed negative attitudes of dissatisfaction or extreme dissatisfaction. Nearly half of LTRs were unsatisfied with the assistance from different sources. There were significant differences in LTRs' AS across assistance sources, with the lowest level of satisfaction reported for self-assistance (*M* = 2.153). Specifically, 57.175% of interviewees were unsatisfied with the results of self-assistance. Interviewees' MAS (*M* = 3.990) was far higher than their SAS, presenting the trend: SAS < EAS < WAS < GAS < MAS. Furthermore, 99.480% of LTRs were affected by negative mood, and 48.550% of LTRs indicated that they could not accept their existing life condition.

**Table 3:** Descriptive statistical analysis of LTRs' AS and its dimensions, NA, and self-acceptance.

**Variance**	***N***	**Minimum**	**Maximum**	**Means**	**Standard deviation**	**Value** **<** **3 (%)**	
AS	1345	1.31	4.80	3.052	0.808	582	43.271
GAS	1345	2.00	5.00	3.670	0.885	214	15.911
EAS	1345	1.00	5.00	2.640	1.533	717	53.309
MAS	1345	1.00	5.00	3.990	1.126	194	14.424
WAS	1345	1.00	5.00	2.810	1.489	535	39.777
SAS	1345	1.00	5.00	2.153	1.277	769	57.175
NA	1345	1.00	5.00	3.650	0.438	1338	99.480
Self-Acceptance	1345	1.00	5.00	2.979	1.469	653	48.550

### NA–Self-Acceptance Cross Analysis

We conducted statistical cross analysis of AS and its dimensions, NA, and self-acceptance. Scores ≥3 points were classified as high-NA and high-acceptance; scores < 3 points were classified as low-NA and low-acceptance. Thus, after crossing NA and self-acceptance, four groups were obtained: low–low, low–high, high–low, and high–high. Detailed analysis is as follows.

**Figure 1:**
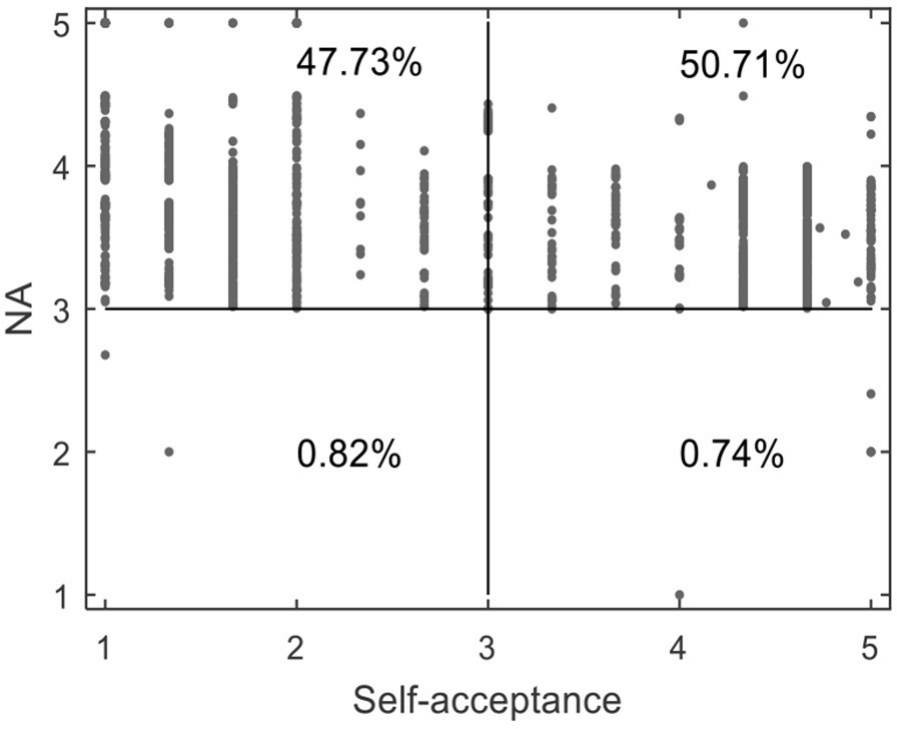
Cross analysis of LTRs' AS and its dimensions, NA, and self-acceptance. Low–low represents NA < 3 and self-acceptance < 3; low–high represents NA < 3 and self-acceptance ≥ 3; high–low represents NA ≥ 3 and self-acceptance < 3; high–high type represents NA ≥ 3 and self-acceptance ≥ 3.

**Figure 2:**
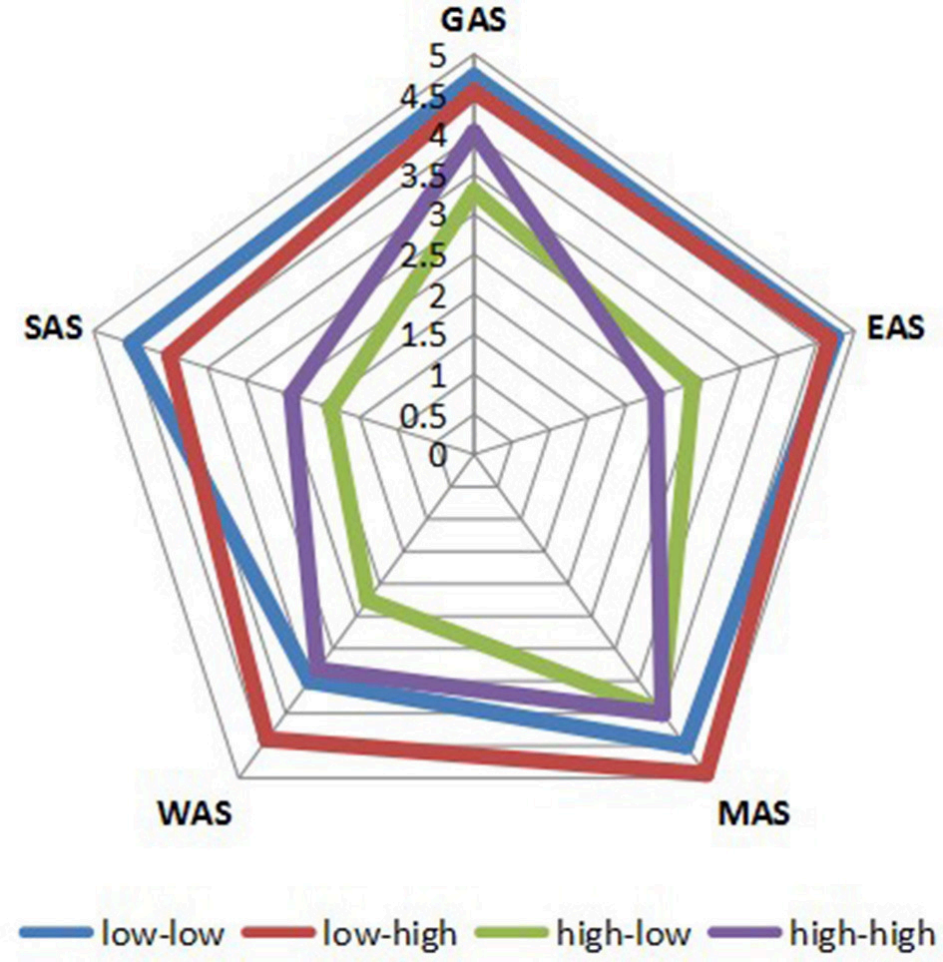
Four groups of LTRs' AS and its dimensions.

As shown in Figure 1, only 0.82 and 0.74% of participants showed low–low and low–high characteristics, respectively; in other words, fewer than 2% of participants reported being unaffected by negative mood. Meanwhile, 47.73% of participants fell into the high–low group. Interestingly, 50.71% of participants reported being affected by negative mood but nonetheless had high self-acceptance (high–high characteristics). As shown in Figure 2, the AS perception of the low–low and low–high groups was far higher than that of the high–low and high–high groups, indicating that NA had a significant negative effect on AS perception. Specifically, GAS and SAS showed the trend of high–low < high–high < low–high < low–low, WAS and MAS showed the trend of high–low < high–high < low–low < low–high, and EAS showed the trend of high–high < high–low < low–high < low–low.

### Distribution Analysis of AS in Bivariate Variables

**Figure 3:**
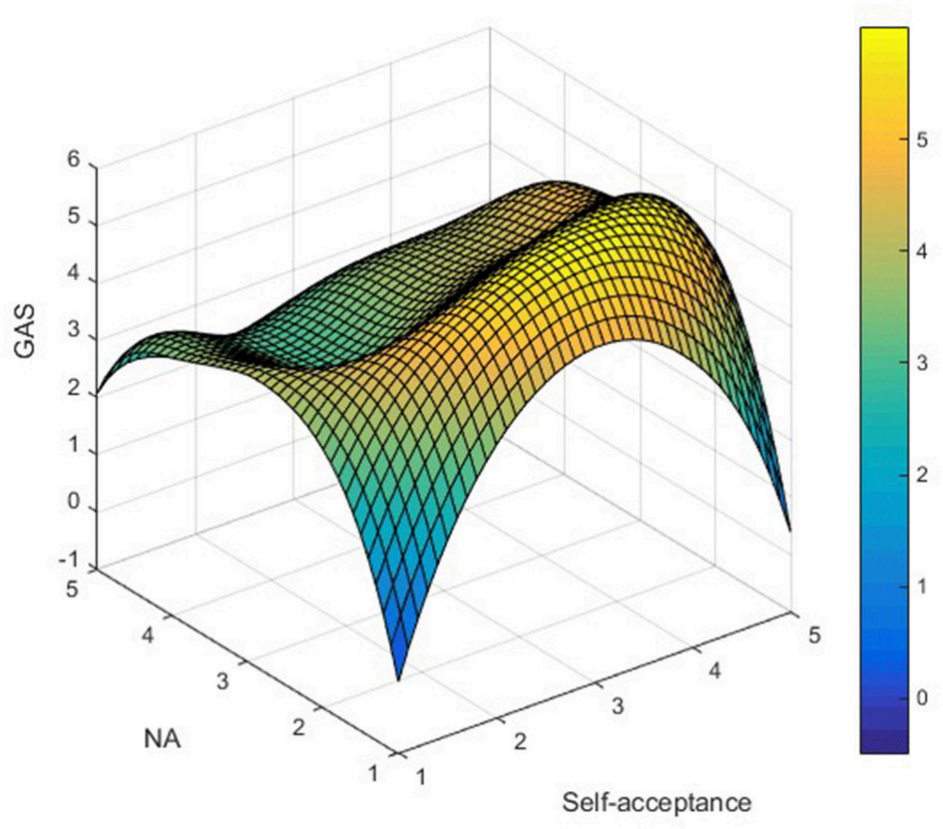
Distribution analysis of GAS.

**Figure 4:**
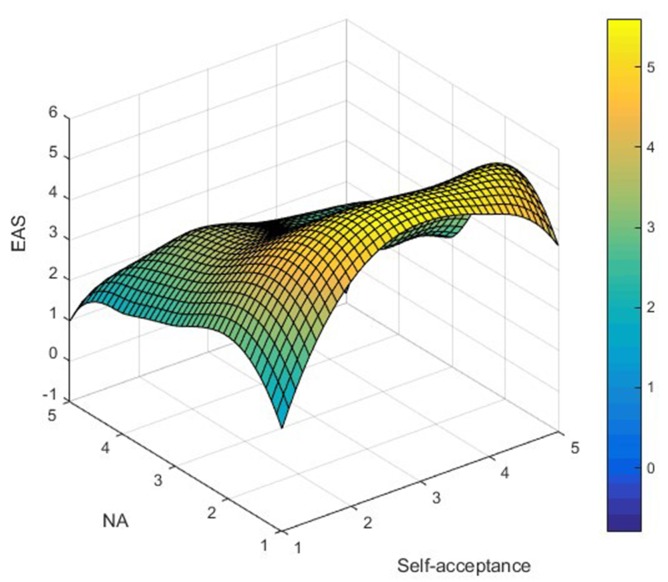
Distribution analysis of EAS.

**Figure 5:**
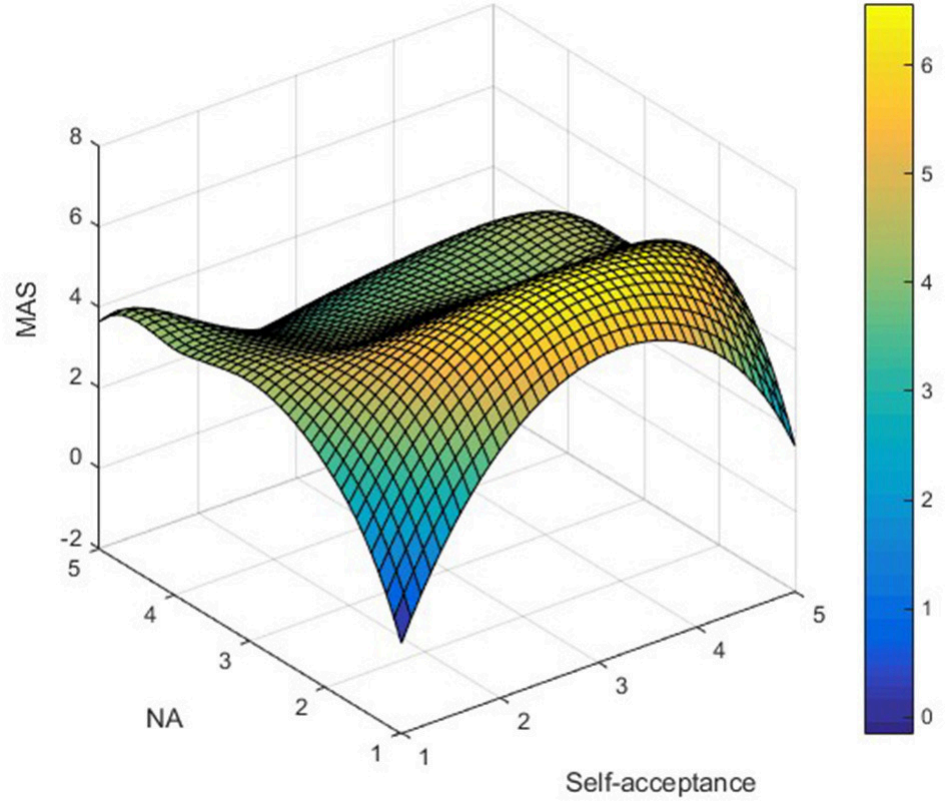
Distribution analysis of MAS.

**Figure 6:**
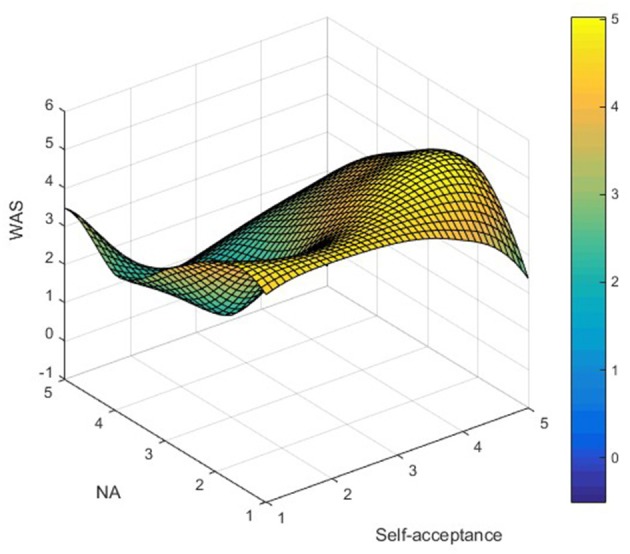
Distribution analysis of WAS.

**Figure 7:**
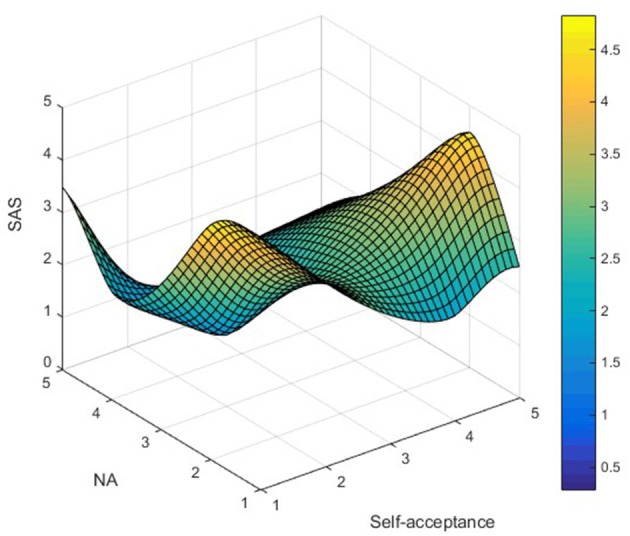
Distribution analysis of SAS.

AS_ij_ was used to represent LTRs' AS simultaneously satisfying NA_i_ (independent variable) and self-acceptance_j_ (moderator) attributes, and Matlab (2008a) software was used to map the data, as shown in Figures 1**–7**. Specifically, *i* = 5 (NA_1_ – NA_5_ representing ratings from “not at all” to “extremely”); j = 5 (self-acceptance_1_-self-acceptance_5_ representing ratings from “strongly disagree” to “strongly agree”).

It can be found from Figure 3 that the apex of LTRs' GAS is mainly distributed in the low–high group, while the low points are distributed in the low–low and low–high groups. In Figure 4, the apex of LTRs' EAS is mainly distributed in the low–high group, while the low points are mainly distributed in the high–low and low-low groups. In Figure 5, the apex of LTRs' MAS is mainly distributed in the low–high group, and the low points are mainly distributed in the low–low and low–high groups. In Figure 6, LTRs' WAS is distributed relatively evenly among the four groups, without showing an obvious trend of concentration. In Figure 7, the apex of LTRs' SAS is mainly distributed in the low–high and low–low groups, and the low points are distributed relatively evenly, without showing an obvious trend of concentration. With the exception of MAS, most of the higher AS were concentrated in the group with low–high characteristics, indicating that when LTRs' negative mood perception is lower and self-acceptance perception is higher, the AS perceived by them is the highest.

### Correlation Analysis

Descriptive statistics for all study variables are shown in Table 4. Across the sample, the average NA level was above the scale midpoints (3.65) and negatively associated with each of the AS ratings. The AS ratings for GAS (3.67), EAS (2.64), MAS (3.99), and WAS (2.81) were above the scale midpoints. The AS ratings' dimensions were also positively with one another, except for EAS and WAS, EAS and SAS.

**Table 4:** Intercorrelations, means, and standard deviations of study variables.

**Variable**	**1**	**2**	**3**	**4**	**5**	**6**	**7**	**8**	**9**	**10**	**11**
(1) Age											
(2) Educational level	−0.297^**^										
(3) Household monthly income	0.052	−0.049									
(4) Number of children	−0.111^**^	−0.007	−0.338^**^								
(5) length of stay	0.015	0.034	−0.076^**^	0.110^**^							
(6) Avg. NA level	−0.043	0.105^**^	0.077^**^	0.036	0.050						
(7) GAS	0.036	0.076^**^	−0.074^**^	−0.005	0.001	−0.402^**^					
(8) EAS	0.044	0.076^**^	−0.086^**^	−0.080^**^	−0.025	−0.265^**^	0.030^*^				
(9) MAS	0.076^**^	0.112^**^	−0.056^*^	−0.118^**^	−0.085^**^	−0.325^**^	0.349^**^	0.098^**^			
(10) WAS	0.004	0.063^**^	−0.061^*^	−0.006	0.019	−0.307^**^	0.686^**^	0.015	0.199^**^		
(11) SAS	−0.023	0.066^**^	−0.067^*^	0.039	0.072^**^	−0.209^**^	0.525^**^	0.030	0.137^**^	0.711^**^	
M	3.40	3.20	3.42	2.57	3.09	3.65	3.67	2.64	3.99	2.81	2.15
SD	0.972	0.772	1.070	1.020	0.793	0.436	0.885	1.533	1.126	1.489	1.277

Avg, average; NA, negative affect.

** p < 0.05;

* *p < 0.1*.

### Cluster Analysis

A hierarchical cluster analysis was performed (*n* = 1,345) to identify the inherent structure of the data (Beckstead, 2002; Ayanore et al., 2016), followed by k-means clustering, which can split the sample in half at random and put the data into clusters (Aldenderfer and Blashfield, 1984). Ward's method with squared Euclidean distance was used to obtain discrete clusters of participants based on z-transformed scores for each of the three perceived AS variables. The agglomeration schedule provided by this method was examined for evidence of the minimum fusion coefficient in the total within-group sum of squares, which indicated distinct clusters (Seiler et al., 2015). Based on these fusion coefficients, two-, three-, and four-cluster solutions were examined. The three-cluster solution (see Table 5) was found to be optimal for two main reasons: (a) Each of the obtained clusters was significantly different from each of the others according to the criterion variables (i.e., AS ratings); (b) the resulting patterns of AS ratings represented understandably meaningful profiles, each of adequate sample size for further statistical analysis.

**Table 5:** Standardized descriptive statistics for clustering variables and demographics by hierarchical cluster member.

**Variable**	**M (SD) (*n* = 259) *Medium***	**M (SD) (*n* = 538) *High***	**M (SD) (*n* = 548) *Low***	***F***	**χ^2^**
GAS	0.014 (0.97)	0.796 (0.54)	−0.788 (0.69)	691.633^***^	
EAS	0.098 (1.05)	−0.033 (0.98)	−0.014 (0.99)	1.585	
MAS	0.142 (0.86)	0.194 (0.95)	−0.257 (1.052)	32.316^***^	
WAS	0.711 (0.42)	0.816 (0.30)	−1.137 (0.31)	5484.827^***^	
SAS	0.745 (0.91)	0.529 (0.87)	−0.871 (0.17)	752.352^***^	
Age					6.510
Educational level					13.775^*^
Household monthly income					11.648
Number of children					7.873
length of stay					1.882
NA	0.195 (1.15)	−0.450 (0.74)	0.350 (0.98)	107.842^***^	
Self-Acceptance	−0.361 (0.87)	0.897 (0.41)	−1.100 (0.30)	1042.056^***^	

Means with different subscripts in each row are significantly different (p < 0.05), as found by Scheffe tests.

*** p < 0.01;

* *p < 0.1*.

The first and smallest cluster (*n* = 259, 19.26%) was featured by medium-level AS ratings, with no highest or lowest scores in AS perception, and therefore was labeled “***medium***.” The second cluster (*n* = 538, 40%) was labeled “***high***” because it was featured by a profile of the highest ratings of GAS (*z* = 0.796), MAS (*z* = 0.194), WAS (*z* = 0.816), and SAS (0.745) indicated by the participants. Specifically, the mean levels of all five AS ratings were at or above 3.61 on a 5-point scale, suggesting an overall profile of relatively high levels of AS perception. Upon closer inspection, the mean levels of GAS was the highest at 4.41, followed by MAS at 4.23. The third and largest cluster (*n* = 548, 40.74%) was labeled “***low***.” The overall profile of this cluster was characterized by the lowest AS ratings for GAS (*z* = −0.788), MAS (*z* = −0.257), WAS (*z* = −1.137), and SAS (−0.871). Specifically, the mean levels of all five AS ratings were at or below 2.28 on a 5-point scale, suggesting an overall profile of relatively low levels of AS perception. Upon closer inspection, the mean level of SAS was the lowest at 1.04, followed WAS at 1.12.

A multiple regression model was adopted to investigate the interaction effects of NA and self-acceptance on three clusters' AS perception. The following regression model was established:

(1) $$Z=\alpha +\alpha_{1}X_{1}+\alpha_{2}X_{2}+\alpha_{1i}X_{1}X_{2}+\mu_{m}$$

In Equation (1), *X*_*m*_*X*_*n*_ represents the interaction effects between two dimensions; for instance, NA × self-acceptance represents the interaction effect between NA and self-acceptance. α_*i*_ is the regression coefficient, α is a constant term, and μ_*m*_ is the error term. In the regression model, we did not initially consider the interaction effect, only considering the main effect of NA (model I), next considering the respective influence of NA and self-acceptance on AS perception (model II), and finally considering correlation and interaction effect between NA and self-acceptance (model III). Before analysis, we centralize all variables. The test results are shown in Table 6.

**Table 6:** Hierarchical regression analysis of three clusters of LTRs.

	**NA (the medium cluster)**			**NA (the high cluster)**			**NA (the low cluster)**		
	**Model 1**	**Model 2**	**Model 3**	**Model 1**	**Model 2**	**Model 3**	**Model 1**	**Model 2**	**Model 3**
(α)	4.676^***^	4.532^***^	5.557^***^	3.703^***^	4.600^***^	3.364^**^	5.476^***^	5.620^***^	5.094
1.Age	0.022	0.019	0.017	0.043^**^	0.041^**^	0.042^*^	0.014	0.018	0.016
2.Educational level	0.014	0.012	0.011	0.130^***^	0.122^***^	0.123^***^	0.005	−0.002	−0.001
3. Household monthly income	−0.067	−0.067^**^	−0.068^**^	−0.060^**^	−0.056^**^	−0.056^**^	−0.025^*^	−0.027^*^	−0.025^*^
4.Number of children	−0.017	−0.013	−0.013	−0.053^**^	−0.049^**^	−0.049^*^	−0.027^*^	−0.031^*^	−0.031^*^
5.length of stay	−0.029	−0.028	−0.030	0.023	0.037	0.037	−0.038^*^	−0.042^*^	−0.040^*^
NA	−0.242^***^	−0.228^***^	−0.500^**^	−0.116^**^	−0.099	0.268	−0.785^***^	−0.764^***^	−0.632^***^
Self		0.067	−0.843		0.230^***^	0.062		0.077^***^	0.183^***^
NA^*^Self			0.249			−0.088			−0.067^***^
R	0.327	0.332	0.338	0.253	0.372	0.375	0.717	0.745	0.746
R^2^	0.107	0.110	0.114	0.064	0.138	0.140	0.514	0.555	0.556
Adj. R2	0.085	0.086	0.086	0.054	0.127	0.127	0.509	0.549	0.550
F	5.013	4.450	4.025	6.066	12.162	10.799	95.415	96.071	84.452
Sig	0.000	0.000	0.000	0.000	0.000	0.000	0.000	0.000	0.000
AS (M, SD)	3.524 (0.458)			3.614 (0.509)			2.278 (0.483)		
NA (M, SD)	3.731 (0.502)			3.449 (0.326)			3.881 (0.429)		
Self (M, SD)	2.450 (1.275)			4.299 (0.607)			1.364 (0.442)		
NS (M, SD)	9.355(5.090)			14.832 (2.505)			5.032 (1.562)		

Avg, average; NA, negative affect.

*** p < 0.01;

** p < 0.05;

* *p < 0.1*.

At the 0.01 significance level, in model I, the main effect of NA on overall AS perception was significant and negative; in model II, self-acceptance had a significantly positive effect on the AS perception of the *high* and *low* clusters, without significant effect on those in the *medium* cluster. In model III, self-acceptance had a significant moderating effect on the relationship between NA and AS among the participants in the *high* and *low* clusters; notably, the interaction effect of NA and self-acceptance exerted a significant negative effect on the main effect of NA on AS perception.

To test our hypothesis that cluster membership and self-acceptance would be associated with differential levels of NA, we used the following multilevel model:

(2) $$\begin{array}{l} \beta_{0j}=\gamma_{00}+\gamma_{01}\left(Age_{j}\right)+\gamma_{02}\left(Education_{j}\right)+\gamma_{03}\left(Income_{j}\right)\\ \;+\gamma_{04}\left(Children_{j}\right)+\gamma_{05}\left(Length_{j}\right)+\gamma_{06}\left(NA_{j}\right)\\ \;+\gamma_{07}\left(Self-accep\tan ce_{j}\right)+\gamma_{08}\left(High_{j}\right)+\gamma_{09}\left(Low_{j}\right)\\ \;+\gamma_{10}\left(Self-accep\tan ce\times High_{j}\right)\\ \;+\gamma_{11}\left(Self-accep\tan ce\times Low_{j}\right)+u_{0j} \end{array}$$

As shown in Equation (2), (γ_00_) AS perception is predicted as a function of demographic characteristics (γ_01_ − γ_05_), grand-mean centered levels of NA across the three clusters (γ_06_), and grand-mean levels of self-acceptance (γ_07_). Additionally, two dummy-codes were created to examine clusters of high (γ_08_) and low (γ_09_); while the medium cluster served as the reference group for these two groups. Finally, the interaction effect terms (γ_10_andγ_11_) between self-acceptance and such dummy-coded groups were added as predictors, and a between-person error term was included (*u*_0*j*_).

An OLS hierarchical regression was performed to explore out hypothesis that cluster membership and self-acceptance would be closely correlated with NA. Specifically, in the first step, the demographic control variables were added, followed by the effects of self-acceptance; in the second step, the two dummy-coded cluster variables were added; finally, the interaction effect terms (cluster variables × self-acceptance) were added into statistical analysis. Each of the mentioned predictors was grand mean centered.

As shown in Table 7, the results indicated that cluster membership and self-acceptance could both significantly predict AS perception, as expected, on the condition that demographic variables and the average NA level were controlled. The change in *R*^2^ indicated statistical moderation, and the significant interaction terms emerged at step 3. Simple slopes tests indicated that at low levels of self-acceptance, both the low (*b* = −0.294, *SE* = 0.20, *p* = 0.310) and high (*b* = −0.089, *SE* = 0.017, *p* = 0.08) clusters showed no significant differences compared with the medium cluster. However, at high levels of self-acceptance, the magnitude of AS perception was higher than that in the medium cluster: *b* = −0.264, *SE* = 0.055, *p* < 0.001 for the low cluster; *b* = −0.108, *SE* = 0.067, *p* < 0.01 for the high cluster.

**Table 7:** OLS hierarchical regression with predictors of negative affect.

**Predictors**	**Coefficient**	**SE**	**SR**	**Significance**
Age	0.080	0.029	0.075	0.005
Educational level	0.137	0.036	0.130	0.000
Household monthly income	−0.124	0.027	−0.114	0.000
Number of children	−0.108	0.030	−0.094	0.000
Length of stay	−0.011	0.034	−0.011	0.686
**STEP 1 CHANGE IN** ***R***^**2**^ **=** 0.043^***^				
NA (X)	−0.346	0.024	−0.326	0.000
*high*	−0.300	0.047	0.280	0.000
*low*	−0.081	0.039	0.080	0.000
**STEP 2 CHANGE IN** ***R***^**2**^ **=** 0.200^***^				
high^*^self-acceptance	−0.209	0.053	0.163	0.000
low^*^self-acceptance	0.108	0.052	−0.080	0.000
**STEP 3 CHANGE IN R**^**2**^ **=** 0.033^***^				

For between-cluster comparisons, Medium was the reference group.

*** p < 0.01;

* *p < 0.1*.

## Discussion

From the perspective of individual variables, this study found that LTRs with higher levels of education are more likely to have higher levels of AS perception. Many scholars have identified a positive and significant association between education and self-rated life satisfaction (Easterlin, 2001; Graham and Pettinato, 2002; Blanchflower and Oswald, 2004). Furthermore, Powdthavee (Powdthavee et al., 2015) argued that education can improve a person's overall quality of life and subjective well-being. We believe that LTRs with a higher level of education have better knowledge of social assistance modes, channels, and the scope of rights of each governing source, more easily understand phenomena such as absence or insufficiency of policy, and more easily perceive the solicitude and support from each social assistance source; thus, they tend to perceive higher AS. From the perspective of household variables, LTRs with lower levels of household monthly income tend to perceive higher-level AS, which is not completely consistent with the results of the prior research on the relationship between satisfaction and comparison income. Specifically, Boyce et al. (2000) held the view that the ranked position of an individual's income can influence the satisfaction judgement. Furthermore, Clark and Oswald (1996) argued that individuals with higher earnings than the comparison level were more likely to feel economically relaxed and relatively happier, thus improving the self-reported satisfaction ratings. However, this could be explained that the participants in our study are a special disadvantaged group because they lost their own earning capacity due to work-related injury, which cause their economic income level is far below the per capita income. Therefore, our research speculates social subsidies may become the only hope of survival for this group, and in this case, the LTRs are eager to obtain help and care soured from social assistance. A point should be noted that these LTRs could be easily satisfied when they receive social assistance compared with the healthy counterparts. Thus, the LTRs with lower income tend to report higher levels of AS perception.

The current study found considerable evidence of the significant association between NA and the five dimensions of AS perception. As predicted, the *high* profile reported the lowest NA levels, while the *low* cluster of participants presented the highest NA levels. In addition, the participants in the *high* profile showed significantly more favorable affective adjustment than those in the *medium* profile. On the contrary, those in the *low* profile were significantly less effective than those in the *medium* profile. Specifically, NA levels were highest in the *low* profile, and AS perception was highest in the low–high (low-NA, high self-acceptance) group, indicating that NA level was a robust predictor of AS in the low cluster. More importantly, the results showed that self-acceptance moderated the relationship between cluster membership and NA levels—that is, higher levels of self-acceptance buffered the negative impact of NA associated with participants' AS perception. Thus, self-acceptance can mitigate the negative effect of LTRs' NA levels on their AS perception. This finding supports the growing literature suggesting that strong self-acceptance can serve a moderating role by promoting people's psychological well-being and thereby positively affecting satisfaction judgments (Abd-Al-Atty et al., 2010; Hu et al., 2012; Xu et al., 2016). Future studies might explore whether improving the level of self-acceptance as a psychological resource can improve LTRs' overall life satisfaction and even their overall quality of life. This would be an important expansion of recent evidence suggesting that positive psychological adjustment can improve individuals' satisfaction judgments.

Through one-way analysis of variance based on the individual and household levels, we found that education and income closely correlates with AS perception. Specifically, the LTRs with higher levels of education and lower levels of household monthly income tend to perceive higher AS perception. In addition, our study also found that the significant association exists between NA and the five dimensions of AS. Specifically, the high profile reported the lowest NA levels, while the low cluster of participants presented the highest NA levels. Furthermore, the results showed that higher levels of self-acceptance buffered the negative impact of NA associated with participants' AS perception. We compare such results with existing literature in similar field, suggesting that the results can be supported by the theoretical basis and empirical evidence from prior studies.

## Conclusion

1. Currently, the AS perception of Chinese LTRs is at an average level, showing an overall trend of SAS < EAS < WAS < GAS < MAS in terms of specific social assistance dimensions. Notably, 85.576% of interviewees have positive evaluations of the assistance received from medical institutions, indicating that medical assistance plays a positive role in the practical work of occupational safety and health. In contrast, 57.175% of LTRs are unsatisfied with the results of self-assistance, with an average of 2.2, suggesting that managers should widen the channel of safeguarding legal rights and establish sound and efficient laws and regulations for LTRs.
2. We found that 99.480% of Chinese LTRs are affected by negative mood. This implies that NA is one of the ubiquitous and non-negligible factors negatively influencing LTRs in their daily life. This indicates that managers should closely observe LTRs' emotional changes, correct their incorrect understanding of the evaluation of self-worth, guide them to make full use of social capital in the curative process and adopt positive coping methods, so as to eliminate or mitigate negative emotions, enhance their self-esteem, confidence, and sense of security, and prompt them to treat their personal condition correctly, cooperate with the treatment positively, and thus improve their AS perception and psychological health in practical ways.
3. The overall AS perception of Chinese LTRs varies significantly with education level and household monthly income; specifically, LTRs with lower levels of education and higher levels household monthly income tend to perceive lower levels of AS perception. This implies that mangers should implement targeted policies and classification management strategies in accordance with changes in the characteristics of LTRs and attach greater importance to those who have lower levels of education and higher levels of income because this group of LTRs tends to report lower levels of perceived AS.
4. Based on cluster analysis, we divided Chinese LTRs into 3 groups: medium (19.26%), high (40%), and low (40.74%) clusters. The high cluster has the highest overall AS perception and the highest WAS and GAS. The low cluster has the lowest overall AS perception, especially in terms of WAS. This indicates that the differences in AS perception could be formed due to various reasons and the overall AS perception of LTRs is closely related to the reported score of WAS. This can prompt managers to identify differences in perceived AS of Chinese LTRs in practical settings and designing targeted strategies. In the meantime, they should attach greater importance to the influence of social assistance from welfare organization on LTRs' AS perception, and properly establish and develop welfare organizations to offer social assistance for LTRs.
5. NA level can serve as a robust predictor of Chinese LTRs' overall AS and satisfaction with the assistance received from five sources of social support. Specifically, NA levels can exert a significant negative impact on the AS perception of Chinese LTRs. More importantly, individual differences in self-acceptance can moderate how cluster membership relates to AS perception. Consistent with our hypothesis, a greater sense of self-acceptance appeared to mitigate the negative effects of NA on the participants in the low cluster. This indicates that managers should pay more attention to the influence of self-acceptance as a psychological resource on LTRs' AS perception, and this would provide theoretical evidence and practical foundation for advancing the governance process of Chinese LTRs' social assistance and improving the relevant practical management work.

## Author Contributions

XH, HC, and SL conceptualization. XH and HC data collection. XH and HC formal analysis. XH writing-original draft. XH and HC writing- review and editing.

### Conflict of Interest Statement

The authors declare that the research was conducted in the absence of any commercial or financial relationships that could be construed as a potential conflict of interest.
